# The Address of the Saturday Fund Chairman

**Published:** 1892-02-27

**Authors:** 


### Passing Topics.

### THE ADDRESS OF THE SATURDAY FUND CHAIRMAN.

The "annual address" delivered by the Chairman of the Hospital Saturday Fund to the Board of Delegates for 1891 is an interesting and instructive publication. Its issue is said to mark the revival of an old practice adhered to in the early days of the Fund, but it wears an air of novelty, and we do not remember that any report so fully stored with information in detail has been previously given to the public.

Mr. Acland wisely announces at the outset a desire to avoid as far as possible topics of a controversial nature, by which we understand him to mean topics likely to lead to controversy among those he is particularly addressing rather than the world at large, because he soon proceeds to embark upon an examination of the relative expenses of the administration of the Sunday and Saturday Funds, which is a very controversial subject indeed, and sure to call forth wide divergences of views. Comparisons, we know, are never welcome, and if fallacies may be upheld in unimpeachable fashion by means of carefully authenticated statistics, it is equally certain that by comparing matters which are not comparable, and overlooking essential differences in the conditions, very inaccurate conclusions may be placed on record.

Mr. Acland has satisfied himself that the cost of administering the Sunday Fund is greater than the cost of the Saturday Fund, but ordinary minds are led by a contemplation of his figures to a very different conclusion. The Sunday Fund collected in 1891 a total of £45,300 at a cost of £1,700. The Saturday Fund collected in the same year less than £20,000, at a cost apparently of £2,800. Mr. Acland does not deal with the figures in this simple way. He argues that because each single collection—no matter what its amount—cost in the case of the Sunday Fund £1, while in the case of the Saturday Fund—no matter again what its amount—it cost 10s. 8d., therefore notwithstanding the larger aggregate collected by the Sunday Fund, and its smaller aggregate of expenditure, it is the more costly of the two—surely a most faulty contention. No fair man would deny that the collection of the Saturday Fund involves an amount of labour out of all proportion to its volume, and it is only right that should be taken into account, but to put forward a calculation of cost which takes no heed of the value of the article obtained in exchange for the expenditure is astoundingly misleading. In that way you have only to multiply and attenuate the collections sufficiently to be landed in a position so paradoxical that the expense of collection will be found to have diminished in the ratio of its increase, and when it reaches vanishing point it will have become equal to the total collected.

That is a sum we will leave the Saturday Fund authorities to work out at their leisure, and if we have any further criticism to offer upon the Chairman's pleasant and generally conciliatory address, it is that we still find a tendency towards the old undue exaltation of the Fund, and the old inability to realise that the assistance the working classes render by its means is but a drop in the ocean of expenditure incurred chiefly for their own relief. We fear we cannot admit that the commercial instincts Mr. Acland does not like to be reminded of have altogether evaporated, but we frankly acknowledge they are not so prominently displayed as heretofore, and it is perfectly true that grants have been made to some institutions of which no direct return has been demanded. This is a welcome indication of an abatement of pretensions which to all hospital workers have appeared so ridiculous, and we are glad to note that the improvement seems to have set in concurrently with Mr. Acland's assumption of office. The working man who has begun to contribute sixpence in the pound towards an expenditure of which three-fourths are incurred in his own or his family's behalf, ought not to be very assertive of his "rights." Some people, and not casuists either, would have little difficulty about still bringing him in a debtor.

